# Age- and Sex-Related Patterns in Coronary CT Angiography Referrals and Coronary Atherosclerotic Burden: A Single-Center Real-World Referral-Based Analysis

**DOI:** 10.3390/medicina62071416

**Published:** 2026-07-22

**Authors:** Andra-Maria Barota-Bebeșelea, Mihai Octavian Negrea, Bogdan Neamtu, Minodora Teodoru, Ciprian Radu Sofariu, Doru-Florian Cornel Moga, Ioan Manitiu, Adriana-Lavinia Cioca

**Affiliations:** 1 “Dr. Alexandru Augustin” Military Emergency Clinical Hospital, 550024 Sibiu, Romania; andra.bebeselea@gmail.com (A.-M.B.-B.); cornel.moga@ulbsibiu.ro (D.-F.C.M.); 2 Clinical Medical Department, Faculty of Medicine, “Lucian Blaga” University, 550024 Sibiu, Romania; bogdan.neamtu@ulbsibiu.ro (B.N.); minodora.teodoru@ulbsibiu.ro (M.T.); ioanmanitiu@yahoo.com (I.M.); adrianalavinia.cioca@ulbsibiu.ro (A.-L.C.); 3 Sibiu Municipal Hospital, 550169 Sibiu, Romania; 4 Department of Clinical Research, Pediatric Clinical Hospital Sibiu, 550166 Sibiu, Romania; 5 County Clinical Emergency Hospital of Sibiu, 550245 Sibiu, Romania; 6 Department of Radiology, Pediatric Clinical Hospital Sibiu, 550166 Sibiu, Romania; ciprianradusofariu@gmail.com; 7 Surgical Clinical Department, Faculty of Medicine, “Lucian Blaga” University, 550024 Sibiu, Romania

**Keywords:** coronary computed tomography angiography, coronary artery disease, coronary artery calcium score, sex differences, chronic coronary syndromes

## Abstract

*Background and Objectives*: Coronary computed tomography angiography (CCTA) has become a central non-invasive imaging modality for the evaluation of suspected chronic coronary syndromes. Nevertheless, referral patterns and sex-related differences in real-world CCTA utilization may vary according to demographic structure, healthcare accessibility, and regional clinical practice. The present study aimed to evaluate age- and sex-related patterns in CCTA referrals and coronary atherosclerotic burden within a real-world Eastern European single-center CCTA referral cohort. *Materials and Methods*: We performed a retrospective analysis of 2742 consecutive CCTA examinations at a tertiary center in Sibiu, Romania, between March 2019 and January 2025. The demographic profile of the referral cohort was compared with the adult population structure of Sibiu County. Subsequently, an age- and sex-stratified proportional subsample of 397 cases was selected from the CCTA cohort for detailed analysis of sex-related differences regarding coronary artery calcium score (CACS), significant coronary stenoses, and significant cardiovascular event risk. *Results*: The age distribution of the CCTA referral cohort differed significantly from that of the adult county population (*p* < 0.01), with referrals concentrated predominantly between 55 and 79 years of age. Female patients were overall more frequently represented within the referral cohort compared with the underlying county population; however, males demonstrated significantly higher coronary artery calcium scores, a greater prevalence of significant coronary stenoses, and higher revascularization risk compared with females—particularly between 50 and 79 years of age—within the stratified proportional subsample. In contrast, patients younger than 40 years demonstrated very low rates of significant coronary disease regardless of sex. *Conclusions*: This study provides a real-world perspective on age- and sex-related patterns in CCTA utilization and coronary atherosclerotic burden within an Eastern European imaging center. The findings highlight the interaction between demographic structure, guideline-directed diagnostic strategies, and coronary imaging utilization in routine clinical practice.

## 1. Introduction

Coronary Artery Disease (CAD) remains one of the leading causes of morbidity and mortality worldwide, representing a major public health burden despite significant advances in prevention, diagnosis, and treatment [1,2,3,4]. Beyond its clinical impact, CAD generates substantial healthcare costs through repeated hospitalizations, long-term medical therapy, invasive procedures, and productivity loss [5,6,7,8]. Consequently, establishing efficient diagnostic pathways capable of accurate risk stratification and appropriate allocation of diagnostic resources is essential in contemporary cardiovascular practice.

Current diagnostic strategies for suspected chronic coronary syndromes rely on integrated clinical risk assessment models designed to estimate the probability of obstructive CAD and guide further investigations. The 2024 guidelines of the European Society of Cardiology for chronic coronary syndromes recommend the use of the Risk Factor-weighted Clinical Likelihood (RF-CL) model, which incorporates age-, sex-, symptom-, and cardiovascular risk factor-related variables to improve estimation of obstructive CAD probability and facilitate selection of subsequent diagnostic testing [9]. Such approaches aim to optimize diagnostic efficiency while minimizing unnecessary investigations and associated healthcare expenditures.

Nevertheless, sex-related differences in the prevalence, presentation, diagnostic evaluation, and management of CAD continue to represent a major challenge in clinical practice [10,11,12]. Women frequently present with atypical symptoms, different patterns of coronary involvement, and a lower prevalence of obstructive epicardial disease despite persistent ischemic symptoms, factors that may contribute to delayed diagnosis or under-recognition [13,14,15,16]. In addition, cardiovascular risk patterns in women evolve across the lifespan, with menopausal transition contributing to increased long-term cardiovascular risk and potentially influencing CAD presentation across different age groups [17]. In contrast, men more commonly exhibit typical obstructive patterns and are often referred earlier for advanced diagnostic investigations. These disparities may ultimately influence both diagnostic yield and therapeutic management [10,18,19,20]. Similar findings were also observed in our previous work evaluating sex-related differences in patients with non-ST elevation acute coronary syndromes [18].

Importantly, sex-related differences in CAD assessment may be further influenced by regional and socioeconomic particularities, including standards of living, cardiovascular risk factor burden, healthcare accessibility, urban–rural distribution, and cultural or behavioral determinants. In addition, considerable geographic variability exists regarding the availability and utilization of non-invasive imaging modalities for CAD diagnosis [21,22,23]. The 2024 European Society of Cardiology guidelines emphasize coronary computed tomography angiography (CCTA) as the preferred first-line non-invasive imaging modality for many patients with suspected obstructive CAD because of its high diagnostic accuracy and negative predictive value. By comparison, although exercise electrocardiography has a more limited role because of its lower sensitivity and specificity, the guidelines still acknowledge its utility in healthcare systems where advanced imaging techniques are less accessible, reflecting the reality of regional disparities in resource availability [9].

Within this context, evaluating local referral patterns and sex-related disparities in patients undergoing CCTA may provide important real-world insights regarding diagnostic behavior and disease characterization. Such analyses are particularly relevant in regions where demographic structure, healthcare infrastructure, and investigation availability may influence patient selection for advanced imaging.

Considering these local specificities, the present study aims to propose and implement a protocol for evaluating the demographic and clinical profile of patients referred for CCTA in a medium-sized Eastern European city, using Sibiu as a real-world model. The study is based on two complementary objectives:

(A) Assessment of the demographic structure of CCTA referrals in one of the main institutions in Sibiu capable of performing this investigation, using the general adult population structure of Sibiu County as a contextual demographic benchmark and interpreting the findings in the context of current clinical guidelines; and

(B) Selection of an age- and sex-stratified proportional subsample from patients referred for CCTA, followed by evaluation of sex-related differences regarding coronary calcium score, major coronary event risk category according to calcium score, the presence of significant coronary lesions, and age distribution.

Through this approach, the study seeks to explore potential sex disparities in the diagnostic pathway of CAD within a local real-world cohort.

## 2. Materials and Methods

### 2.1. Study Setting and Population

The present study was conducted through a retrospective analysis of data collected from patients evaluated by coronary computed tomography angiography (CCTA) at the Sibiu Clinical Pediatric Hospital between 18 March 2019, and 15 January 2025. During this period, there were a total of 2742 CCTA examinations within the institution.

The study was designed in two consecutive stages.

First, the demographic profile of patients referred for CCTA was analyzed and compared with the demographic structure of the general adult population of Sibiu County. Comparisons were performed according to age categories and sex distribution in order to evaluate potential disparities in referral patterns for advanced coronary imaging. The 2023 adult Sibiu County population was used as the reference demographic structure for comparison with the CCTA referral cohort. Demographic data were obtained from the data published by the Sibiu County Directorate of Statistics in 2024 [24]. Similar demographic stratification approaches have previously been used in Romanian epidemiologic studies [25,26]. Because the referral cohort covered March 2019 to January 2025, this comparison was considered an approximation of demographic representativeness rather than a direct incidence-rate analysis.

Second, a stratified age- and sex-proportional subsample was extracted from the overall CCTA cohort. Because detailed imaging variables required manual review of individual coronary CT angiography examinations, these data were collected in a an age- and sex-stratified subsample rather than in the entire cohort of 2742 referrals. Patients were stratified by age group and sex, and selected alphabetically within each stratum to preserve the demographic structure of the source cohort. Given the exploratory nature of the study, this sampling approach was considered appropriate.

Sample size estimation for the stratified proportional subsample was performed using the standard finite population correction formula assuming a 95% confidence level, 5% margin of error, and maximal variance assumption (*p* = 0.5) according to Equation (1):

(1) $$n=\frac{N\times Z^{2}\times p(1-p)}{d^{2}\left(N-1\right)+Z^{2}\times p(1-p)}$$

where

- *n* = required sample size;
- *N* = population size;
- *Z* = Z-score corresponding to the selected confidence level;
- *p* = estimated population proportion;
- *d* = margin of error.

Using:

- *N* = 2742 (CCTA referral cohort);
- *Z* = 1.96 (for a confidence level of 95%);
- *p* = 0.5 (estimated for sex distribution);
- *d* = 0.05.

This yielded a minimum representative sample size of approximately 338 individuals. To preserve adequate subgroup sizes for stratified proportional sampling and subsequent subgroup analyses, adjacent age categories were merged into broader age intervals (<40, 40–49, 50–59, 60–69, 70–79, and ≥80 years). The subsample was stratified according to both age interval and sex distribution within each age category relative to the overall CCTA referral cohort.

A final stratified proportional subsample of 397 patients was subsequently selected to preserve proportional representation across demographic strata.

Within this subgroup, demographic and imaging-related parameters associated with coronary artery disease were collected and analyzed. The evaluated variables included coronary artery calcium (CAC) score, Agatston score–based coronary event risk categories, presence or absence of coronary lesions, as well as lesion number and severity.

Significant coronary lesions were analyzed according to predefined severity thresholds. A coronary lesion was predefined as obstructive (significant) when the diameter stenosis was ≥50% in the left main coronary artery or ≥70% in any other major epicardial vessel (LAD—left anterior descending coronary artery, LCX—left circumflex coronary artery, RCA—right coronary artery) and their main branches with a reference diameter ≥2 mm. These cut-offs are concordant with the 2024 ESC (European Society of Cardiology) Guidelines on chronic coronary syndromes [9], the 2021 ACC/AHA/SCAI (American College of Cardiology/American Heart Association/Society for Cardiovascular Angiography & Interventions) revascularization guideline [27], and the CAD-RADS (Coronary Artery Disease Reporting and Data System) 2.0 reporting system [28], and have been validated against invasive coronary angiography and fractional flow reserve in landmark trials [29,30,31,32,33]. Stenosis of 1–49% was classified as nonobstructive; chronic total occlusions were coded as 100% stenosis.

Furthermore, in addition to the continuous coronary artery calcium score (CACS), patients were classified according to Agatston score–based coronary event risk categories, as reported in their original CCTA examination, using cut-off values previously established in the literature. In particular, a CAC score >400, as determined by the Agatston method, was used to define a significant risk of having a cardiovascular event [34].

Subsequently, sex-related differences were assessed regarding CAC score distribution, Agatston-derived cardiovascular event risk category, prevalence of significant coronary lesions, and age distribution.

### 2.2. Statistical Analysis

Categorical variables were described using frequencies and percentages. Continuous variables were described using measures of central tendency and dispersion, including mean values, standard deviations, minimum and maximum values, interquartile ranges, and 95% confidence intervals where applicable.

The distribution of continuous variables was evaluated for normality using the Shapiro–Wilk or Kolmogorov–Smirnov tests, depending on sample characteristics. Comparative analyses between groups were subsequently performed using parametric or non-parametric statistical tests as appropriate. Associations between categorical variables were assessed using Pearson’s chi-square test or Fisher’s exact test. To identify specific age categories contributing to the overall distributional difference, adjusted standardized residuals derived from contingency table analysis were examined.

Continuous variables were compared using the independent samples t-test or Mann–Whitney U test.

A two-sided *p*-value < 0.05 was considered indicative of statistical significance throughout the analysis.

Generative AI (ChatGPT, OpenAI, GPT-5.5) was used to refine the language for clarity and improve the readability of the manuscript. AI assistance was not used for data analysis, interpretation of results, or the generation of original scientific content. The final content was reviewed and edited by the authors to ensure accuracy and alignment with the study objectives. The authors take full responsibility for the scientific integrity and conclusions presented in this manuscript.

## 3. Results

### 3.1. Sibiu County Population and Coronary CTA Referrals in the Study Imaging Center

**Figure 1:**
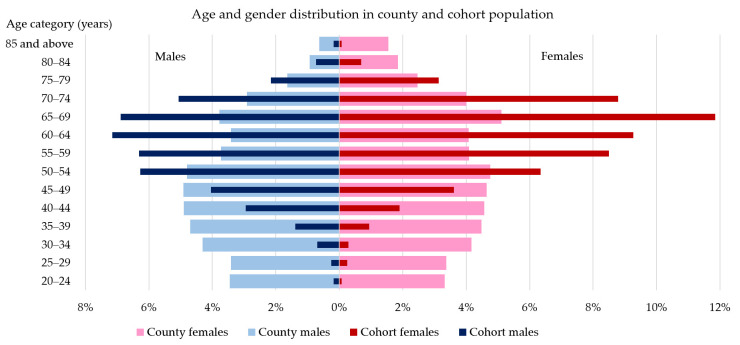
Age- and sex-specific distribution of the Sibiu County population and the coronary CT angiography referral cohort. Bars represent the percentage contribution of each age and sex subgroup to the total population and total referral cohort, respectively. Male categories are displayed on the left side and female categories on the right side of the pyramid.

The age- and sex-specific distributions of the Sibiu county population and the coronary CT angiography referral cohort are presented in Figure 1.

The age distribution of the coronary CT angiography referral cohort differed significantly from that of the adult Sibiu County population (*p* < 0.001). Table 1 presents the proportional age-group distributions of the county population and referral cohort together with the adjusted standardized residual values derived from contingency table analysis.

**Figure 2:**
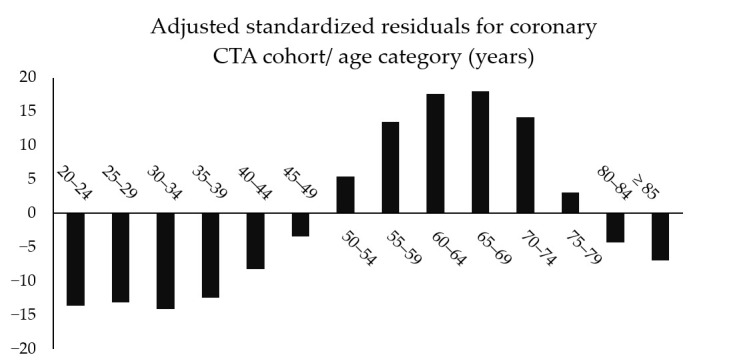
Adjusted standardized residuals for age-group representation within the coronary CT angiography referral cohort relative to the adult Sibiu County population.

Figure 2 provides a visual illustration of the adjusted standardized residuals for age-group representation within the coronary CT angiography referral cohort relative to the adult Sibiu County population.

Sex distribution comparisons across age categories between the adult Sibiu county population and the coronary CTA referral cohort are summarized in Table 2.

### 3.2. Sex-Related Differences Within the CCTA Stratified Proportional Subsample

Table 3 reports the number of patients in the source CCTA cohort and the selected stratified proportional subsample for each age–sex stratum. Due to the relatively low number of patients younger than 40 years, these age categories were analyzed collectively. Similarly, due to the relatively low number of patients older than 80 years, these age categories were also analyzed collectively.

Data regarding sex-related differences in the stratified age-and-sex proportional coronary CCTA subsample are provided in Table 4.

## 4. Discussion

The age distribution of the coronary CT angiography referral cohort differed significantly from that of the adult Sibiu County population (*p* < 0.001). As illustrated by the adjusted standardized residual analysis, age categories between 55 and 79 years contributed most strongly to the observed deviation in cohort structure relative to the county population baseline. The highest positive residual values were observed in the 60–64-year and 65–69-year age groups, indicating a greater proportional contribution of these categories to the CCTA referral cohort.

Conversely, negative residual values were observed predominantly in younger age categories, particularly between 20 and 44 years, reflecting lower proportional representation within the referral cohort relative to the underlying county population structure. Negative residual values were also observed in the 80–84-year and ≥85-year age groups. Relatively few CCTA referrals were observed in patients younger than 35 years or older than 85 years. The age distribution of CCTA referrals observed in our cohort—with a marked under-representation of patients younger than 35 years and older than 85 years—reflects well-established clinical, biological, and guideline-driven determinants of test selection rather than a referral bias per se.

At the lower end of the age spectrum (<35 years), the pre-test probability of obstructive coronary artery disease is intrinsically very low, with reported prevalence of angiographically significant CAD below 5% even in symptomatic populations [35]. Current pre-test probability models endorsed by the 2024 ESC guidelines advise against anatomical imaging when the likelihood of CAD is very low, as positive findings would be dominated by false positives and incidental non-obstructive plaque [9]. In addition, the cumulative lifetime risk of stochastic effects from ionizing radiation is also greatest at younger ages [36,37]. Accordingly, clinical assessment and functional, non-radiating tests are preferred as first-line evaluations in young symptomatic patients, with CCTA reserved for those with a strong suspicion of premature atherosclerosis (e.g., familial hypercholesterolemia, family history of premature CAD, or persistent typical angina).

At the opposite side of the age spectrum (>85 years), the low utilization of CCTA is multifactorial. Diagnostic performance declines because extensive coronary calcification produces blooming artefacts that overestimate stenosis severity and reduce specificity [38,39]; adequate heart-rate control and image quality are harder to achieve given the higher prevalence of atrial fibrillation and contraindications to beta-blockers [40]; and the risk of contrast-induced kidney injury is greater [41]. In this context, complementary functional approaches such as stress myocardial CT perfusion may prove useful in selected patients, although their precise role in routine clinical practice remains under ongoing investigation [42]. Most importantly, management of chronic coronary syndromes in this age group is increasingly conservative—frailty, multimorbidity, and limited life expectancy reduce the expected benefit of revascularization, and thus the added value of an anatomical diagnosis—and both the 2024 ESC and the 2021 ACC/AHA/SCAI guidelines recommend an individualized, frailty-adjusted approach [9,27,43].

Taken together, these considerations explain why the age distribution of CCTA referrals in real-world practice—including in our cohort—follows a characteristic inverted-U shape, concentrating diagnostic activity in the 50–80-year window, where the pre-test probability of obstructive CAD, the diagnostic accuracy of CCTA, and the therapeutic implications of its findings are all maximal.

In our study, sex distribution differed significantly between the adult Sibiu County population and the coronary CTA referral cohort overall (*p* < 0.01), with males accounting for a lower proportion of the referral cohort than of the county population (44.3% vs. 47.5%). Age-stratified analysis demonstrated significant sex distribution differences across multiple age categories. Male patients were significantly more frequent in the CCTA cohort than in the county population in the 30–34-year and 40–44-year age groups, as well as among patients aged above 80 years. Conversely, male patients accounted for a significantly lower proportion of the CCTA cohort than of the county population in the 55–59-year, 65–69-year, and 70–74-year age groups. No statistically significant differences were observed in the remaining age strata.

The observed age-dependent variation in referral patterns likely reflects the interaction between the epidemiology of coronary artery disease, sex-specific clinical presentation, and contemporary referral practices. Women more frequently present with symptoms suggestive of myocardial ischemia despite a lower prevalence of obstructive coronary artery disease and are disproportionately affected by ischemia with non-obstructive coronary arteries—INOCA (Ischemia with No Obstructive Coronary Arteries)/ANOCA (Angina With Non-Obstructive Coronary Arteries), coronary microvascular dysfunction, and coronary vasomotor disorders, all of which may increase referral for CCTA. Conversely, men generally develop coronary atherosclerosis earlier and exhibit a greater burden of obstructive disease, potentially contributing to their greater representation among referrals in selected younger age groups. Similar sex-related differences in the presentation and diagnostic evaluation of coronary artery disease have been reported in recent studies [44,45,46].

Within the proportional age- and sex-stratified subsample, however, males demonstrated significantly higher coronary artery calcium scores (CACS), a greater prevalence of significant coronary stenoses, and a higher prevalence of significant cardiovascular event risk than females. These sex-related differences became progressively more pronounced with advancing age, particularly between 50 and 79 years, where males consistently exhibited higher CACS values and higher frequencies of significant coronary lesions. In contrast, patients younger than 40 years demonstrated very low CACS values and no cases of significant coronary stenoses or significant cardiovascular event risk in either sex. Among patients aged 80 years and above, no statistically significant sex-related differences were identified regarding CACS distribution, significant coronary stenoses, or significant cardiovascular event risk, although the relatively small number of patients in this subgroup likely limited statistical power.

Overall, although women constituted a slightly larger proportion of the referral cohort than expected from the background adult population, male patients within the proportional age- and sex-stratified subsample consistently exhibited a substantially greater coronary atherosclerotic burden. These findings are consistent with the well-established sex-related epidemiology of coronary artery disease, whereby men develop coronary atherosclerosis earlier and generally accumulate a greater burden of obstructive disease throughout middle and older age. Similar sex-related differences in coronary atherosclerotic burden have been consistently reported across diverse populations [10,11,12,47,48,49].

### Strengths, Limitations, and Future Directions

The present study has several limitations. First, it was based exclusively on a retrospective coronary CT angiography imaging database, and only variables routinely documented within imaging records were available for analysis. Standardized information regarding cardiovascular risk factors, clinical presentation, referral indication, medication use, pre-test probability, and the underlying nosological structure of the study population was therefore unavailable. Consequently, the observed age- and sex-related differences in coronary artery calcium burden and stenosis severity should be interpreted as descriptive associations rather than independent or causal relationships, as they likely reflect the complex interplay of unmeasured clinical factors within different demographic groups. Second, detailed plaque characterization was performed on a stratified age- and sex-proportional subsample to enable comprehensive manual image review. This approach substantially reduced the time required for manual extraction of detailed imaging variables compared with reviewing all 2742 referrals. However, because patient selection within each stratum was performed using a deterministic alphabetical procedure rather than probability-based random sampling, the subsample was designed to respect age and sex stratification, and representativeness for imaging findings should be interpreted in this context. Accordingly, within-stratum selection was outcome-blind but non-random, and residual selection bias cannot be excluded.

In addition, because the county demographic data were derived from a single reference year, whereas the CCTA referral cohort was accumulated over multiple years, the findings should be interpreted as a comparison of demographic structure rather than as a true population-based referral-rate analysis. Furthermore, the age-stratified sex comparisons should be regarded as exploratory subgroup analyses and interpreted with appropriate caution.

Despite these limitations, the study has several important strengths. By relying exclusively on routinely collected imaging data, the methodology is simple, readily reproducible, and can be implemented by virtually any CCTA center without dedicated prospective data collection. Rather than establishing causal relationships, this approach provides a practical overview of local referral patterns and coronary imaging findings, allowing centers to identify demographic trends that may warrant further investigation. More broadly, this pragmatic methodology may serve as a reproducible framework for individual CCTA centers to characterize their own referral populations, identify region-specific epidemiological patterns, and generate hypotheses for future clinically enriched investigations. Future studies should integrate standardized imaging, clinical, and cardiovascular risk-factor data across the entire cohort, enabling adjustment for important confounders while providing valuable insight into the local epidemiology of coronary artery disease. Such integrated registries could facilitate the identification of community-specific cardiovascular risk profiles and support the development of targeted prevention strategies and public health interventions.

## 5. Conclusions

The present study provides a real-world perspective on age- and sex-related referral patterns for coronary CT angiography and the distribution of coronary atherosclerotic burden within an age- and sex-proportional stratified subsample of an Eastern European CCTA referral cohort. By contextualizing referral patterns against the demographic structure of the underlying adult county population and integrating imaging-derived markers of coronary disease severity, the study contributes to a better understanding of how contemporary guideline-directed CCTA is applied in routine clinical practice. These findings may serve as a basis for future prospective studies incorporating clinical risk factors and referral indications to further refine patient selection and optimize the use of CCTA in the evaluation of chronic coronary syndromes.

## Figures and Tables

**Table 1:** Comparison of age-group distributions between the adult Sibiu County population and the coronary CT angiography referral cohort.

Age Group (Years)	County (%)	CCTA Cohort (%)	Adjusted Standardized Residual for the CCTA Cohort
20–24	6.78	0.26	−13.6
25–29	6.79	0.51	−13.1
30–34	8.47	0.98	−14.1
35–39	9.18	2.33	−12.4
40–44	9.46	4.85	−8.2
45–49	9.56	7.66	−3.4
50–54	9.56	12.62	5.4
55–59	7.83	14.81	13.5
60–64	7.49	16.41	17.6
65–69	8.89	18.75	18
70–74	6.91	13.86	14.2
75–79	4.11	5.29	3.1
80–84	2.79	1.42	−4.3
85 and above	2.18	0.26	−6.9

Percentages represent the proportional contribution of each age category to the total county population and total referral cohort, respectively.

**Table 2:** Sex distribution across age groups of county and cohort populations.

Age Group (Years)	County Males (%)	CCTA Cohort Males (%)	*p*-Value
ALL	47.5	44.3	<0.01
20–24	51	71.4	0.454 *
25–29	50.3	50.0	1 *
30–34	50.8	70.4	0.042
35–39	51.2	59.4	0.19
40–44	51.7	60.9	0.034
45–49	51.4	52.9	0.673
50–54	50.2	49.7	0.864
55–59	47.7	42.6%	0.041
60–64	45.5	43.6	0.41
65–69	42.5	36.8	<0.01
70–74	42.1	36.6	0.03
75–79	40	40.7	0.87
80–84	33.6	51.3	0.02
85 and above	29.2	71.4	0.026

*p*-values marked with “*” were obtained using Fisher’s exact test. Percentages represent the proportion of males within the county population and the CCTA referral cohort, respectively.

**Table 3:** Age–Sex Stratum Sizes in the Original CCTA Cohort and the Stratified Proportional Subsample.

Age Group (Years)	Original CCTA Cohort Males	Stratified Proportional Subsample Males
ALL	1214 (44.28%)	177 (44.58%)
Under 40	69 (61.61%)	9 (64.29%)
40–49	192 (55.98%)	28 (56.00%)
50–59	345 (45.88%)	50 (46.30%)
60–69	385 (39.94%)	56 (39.72%)
70–79	198 (37.71%)	28 (38.36%)
80 and above	25 (54.35%)	6 (54.55%)

Data are presented as absolute values and percentages within the age stratum.

**Table 4:** Sex-related differences in the stratified proportional CCTA subsample.

Age Group	CACS			Significant Coronary Stenoses			Significant Cardiovascular Event Risk (Agatston Score–Based Risk Category)		
	Males	Females	*p*-Value	Males %	Females %	*p*-Value	Males %	Females %	*p*-Value
ALL	67.3 [360.7]	2.35 [78.75]	<0.01	40.7	19.5	<0.01	19.8	6.8	<0.01
Under 40	0 [3.9]	0 (constant)	0.364	0	0	-	0	0	-
40–49	0.15 [3.93]	0 [<0.001]	0.011	17.9	9.1	0.444 *	3.6	0	1 *
50–59	42.45 [142.33]	0 [2.33]	<0.01	32	13.8	0.023	10	3.4	0.168
60–69	119.45 [426.63]	8 [117.65]	<0.01	53.6	18.8	<0.01	26.8	8.3	<0.01
70–79	339.7 [727.35]	44.7 [153.95]	<0.01	60.7	31.1	0.013	39.3	8.9	<0.01
80 and above	505.08 ± 461.99	244.5 ± 244.26	0.288	66.7	60	1	50	40	1

Results presented for continuous variables as median [IQR] where not normally distributed or mean ± SD (standard deviation) where normally distributed (i.e., in the 80 and above category); *p*-values marked with “*” were obtained using Fisher’s exact test.

## Data Availability

The data presented in this study are available on request from the corresponding author due to privacy and institutional restrictions.

## Abbreviations

The following abbreviations are used in this manuscript:

ACC	American College of Cardiology
AHA	American Heart Association
ANOCA	Angina With Non-Obstructive Coronary Arteries
AV	Atrioventricular
CAC	Coronary Artery Calcium
CAD	Coronary Artery Disease
CAD-RADS	Coronary Artery Disease Reporting and Data System
CACS	Coronary Artery Calcium Score
CCTA	Coronary Computed Tomography Angiography
CT	Computed Tomography
ESC	European Society of Cardiology
EACVI	European Association of Cardiovascular Imaging
ECG	Electrocardiography
IQR	Interquartile Range
INOCA	Ischemia with No Obstructive Coronary Arteries
LAD	Left Anterior Descending Coronary Artery
LCx	Left Circumflex Coronary Artery
MRI	Magnetic Resonance Imaging
RCA	Right Coronary Artery
RF-CL	Risk Factor-Weighted Clinical Likelihood
SCAI	Society for Cardiovascular Angiography & Interventions
SCCT	Society of Cardiovascular Computed Tomography
SD	Standard Deviation

## References

[1] Nedkoff L., Briffa T., Zemedikun D., Herrington S., Wright F.L. (2023). Global Trends in Atherosclerotic Cardiovascular Disease. Clin. Ther..

[2] Heusch G. (2024). Myocardial Ischemia/Reperfusion: Translational Pathophysiology of Ischemic Heart Disease. Med.

[3] Khan M.A., Hashim M.J., Mustafa H., Baniyas M.Y., Al Suwaidi S.K.B.M., AlKatheeri R., Alblooshi F.M.K., Almatrooshi M.E.A.H., Alzaabi M.E.H., Al Darmaki R.S. (2020). Global Epidemiology of Ischemic Heart Disease: Results from the Global Burden of Disease Study. Cureus.

[4] Mensah G.A., Fuster V., Murray C.J.L., Roth G.A., Mensah G.A., Abate Y.H., Abbasian M., Abd-Allah F., Abdollahi A., Abdollahi M. (2023). Global Burden of Cardiovascular Diseases and Risks, 1990-2022. J. Am. Coll. Cardiol..

[5] Rittiphairoj T., Bulstra C., Ruampatana C., Stavridou M., Grewal S., Reddy C.L., Atun R. (2025). The Economic Burden of Ischaemic Heart Diseases on Health Systems: A Systematic Review. BMJ Glob. Health.

[6] Shakya S., Shrestha A., Robinson S., Randall S., Mnatzaganian G., Brown H., Boyd J., Xu D., Lee C.M.Y., Brumby S. (2025). Global Comparison of the Economic Costs of Coronary Heart Disease: A Systematic Review and Meta-Analysis. BMJ Open.

[7] Kotseva K., Gerlier L., Sidelnikov E., Kutikova L., Lamotte M., Amarenco P., Annemans L. (2019). Patient and Caregiver Productivity Loss and Indirect Costs Associated with Cardiovascular Events in Europe. Eur. J. Prev. Cardiol..

[8] Luengo-Fernandez R., Little M., Gray A., Torbica A., Maggioni A.P., Huculeci R., Timmis A.D., Vardas P., Leal J. (2024). Cardiovascular Disease Burden Due to Productivity Losses in European Society of Cardiology Countries. Eur. Heart J. Qual. Care Clin. Outcomes.

[9] Vrints C., Andreotti F., Koskinas K.C., Rossello X., Adamo M., Ainslie J., Banning A.P., Budaj A., Buechel R.R., Chiariello G.A. (2024). 2024 ESC Guidelines for the Management of Chronic Coronary Syndromes. Eur. Heart J..

[10] Al Hamid A., Beckett R., Wilson M., Jalal Z., Cheema E., Al-Jumeily O.B.E.D., Coombs T., Ralebitso-Senior K., Assi S. (2024). Gender Bias in Diagnosis, Prevention, and Treatment of Cardiovascular Diseases: A Systematic Review. Cureus.

[11] Kim H.-L. (2024). Sex Differences in Coronary Atherogenesis: A Narrative Review. Ewha Med. J..

[12] Kim H.-L., Kim M.-A. (2023). Sex Differences in Coronary Artery Disease: Insights From the KoRean WOmen’S Chest Pain REgistry (KoROSE). Korean Circ. J..

[13] Theofilis P., Vlachakis P.K., Mantzouranis E., Sakalidis A., Chrysohoou C., Leontsinis I., Lazaros G., Dimitriadis K., Drakopoulou M., Vordoni A. (2025). Acute Coronary Syndromes in Women: A Narrative Review of Sex-Specific Characteristics. Angiology.

[14] Mehta P.K., Huang J., Levit R.D., Malas W., Waheed N., Bairey Merz C.N. (2022). Ischemia and No Obstructive Coronary Arteries (INOCA): A Narrative Review. Atherosclerosis.

[15] van Oosterhout R.E.M., de Boer A.R., Maas A.H.E.M., Rutten F.H., Bots M.L., Peters S.A.E. (2020). Sex Differences in Symptom Presentation in Acute Coronary Syndromes: A Systematic Review and Meta-analysis. J. Am. Heart Assoc..

[16] El Bassiri Y., Azeem A., Sharma A.C., Hassan M., Hassan M., Omari I. (2025). Gender Disparities in Ischemic Heart Disease Management: Underdiagnosis in Women and Differences in Treatment. Cureus.

[17] Chrysohoou C., Iliakis P., Pitsillidi A., Manta E., Barkas F., Liberopoulos E., Sfikakis P.P., Pitsavos C., Tsioufis C., Panagiotakos D. (2026). Twenty-Year Cardiovascular Disease Incidence in Menopausal Women: Insights from the ATTICA Study. Climacteric.

[18] Negrea M.O., Zdrenghea D., Teodoru M., Neamțu B., Cipăian C.R., Pop D. (2022). Gender Particularities and Prevalence of Atypical Clinical Presentation in Non-ST Elevation Acute Coronary Syndrome. J. Cardiovasc. Dev. Dis..

[19] Marzà-Florensa A., Kiss P., Youssef D.M., Jalali-Farahani S., Lanas F., Di Cesare M., Juanatey J.R.G., Taylor S., Uijl A., Grobbee D.E. (2025). Sex Differences in Acute Coronary Syndromes: A Scoping Review Across the Care Continuum. Glob. Heart.

[20] Pana T.A., Mamas M.A., Myint P.K., Dawson D.K. (2025). Sex Differences in Myocardial Infarction Care and Outcomes: A Longitudinal Scottish National Data-Linkage Study. Eur. J. Prev. Cardiol..

[21] Brown L., Cambron C., Post W.S., Brandt E.J. (2024). The Role of Social Determinants of Health in Atherosclerotic Cardiovascular Disease. Curr. Atheroscler. Rep..

[22] Timmis A., Aboyans V., Vardas P., Townsend N., Torbica A., Kavousi M., Boriani G., Huculeci R., Kazakiewicz D., Scherr D. (2024). European Society of Cardiology: The 2023 Atlas of Cardiovascular Disease Statistics. Eur. Heart J..

[23] Cenko E., Manfrini O., Fabin N., Dorobantu M., Kedev S., Milicic D., Vasiljevic Z., Bugiardini R. (2023). Clinical Determinants of Ischemic Heart Disease in Eastern Europe. Lancet Reg. Health-Eur..

[24] Anuar S.B. (2024). Anuarul JudeȚUlui Sibiu- EdiȚIa 2024.

[25] Gheonea C., Plesca D., Dragomir D., Oraseanu D., Cernatescu I., Nanulescu M., Neamtu M., Bisca N., Chereches-Panta P., Gotia S. (2009). Childhood Asthma Prevalence in Romania: An Epidemiologic Study. D104. Epidemiology of Pediatric Respiratory Diseases.

[26] Dorobantu M., Cojocaru C., Stanciulescu L., Pop C., Bala C., Benedek T., Parepa I., Lighezan D., Darabont R., Darabantiu D. (2023). Ups and Downs of Conducting a National Representative Survey on Hypertension during Pandemic Time: Main Results of SEPHAR IV. J. Hypertens..

[27] Lawton J.S., Tamis-Holland J.E., Bangalore S., Bates E.R., Beckie T.M., Bischoff J.M., Bittl J.A., Cohen M.G., DiMaio J.M., Don C.W. (2022). 2021 ACC/AHA/SCAI Guideline for Coronary Artery Revascularization: A Report of the American College of Cardiology/American Heart Association Joint Committee on Clinical Practice Guidelines. Circulation.

[28] Cury R.C., Leipsic J., Abbara S., Achenbach S., Berman D., Bittencourt M., Budoff M., Chinnaiyan K., Choi A.D., Ghoshhajra B. (2022). CAD-RADSTM 2.0-2022 Coronary Artery Disease-Reporting and Data System. J. Cardiovasc. Comput. Tomogr..

[29] Fearon W.F., Zimmermann F.M., De Bruyne B., Piroth Z., van Straten A.H.M., Szekely L., Davidavičius G., Kalinauskas G., Mansour S., Kharbanda R. (2022). Fractional Flow Reserve–Guided PCI as Compared with Coronary Bypass Surgery. N. Engl. J. Med..

[30] Puymirat E., Cayla G., Simon T., Steg P.G., Montalescot G., Durand-Zaleski I., le Bras A., Gallet R., Khalife K., Morelle J.-F. (2021). Multivessel PCI Guided by FFR or Angiography for Myocardial Infarction. N. Engl. J. Med..

[31] Sakai K., Shin D., Singh M., Malik S., Dakroub A., Sami Z., Weber J., Cao J.J., Parikh R., Chen L. (2025). Diagnostic Performance and Clinical Impact of Photon-Counting Detector Computed Tomography in Coronary Artery Disease. J. Am. Coll. Cardiol..

[32] Mohamed M., Bosserdt M., Wieske V., Dubourg B., Alkadhi H., Garcia M.J., Leschka S., Zimmermann E., Shabestari A.A., Nørgaard B.L. (2023). Combination of Computed Tomography Angiography with Coronary Artery Calcium Score for Improved Diagnosis of Coronary Artery Disease: A Collaborative Meta-Analysis of Stable Chest Pain Patients Referred for Invasive Coronary Angiography. Eur. Radiol..

[33] Schlattmann P., Wieske V., Bressem K.K., Götz T., Schuetz G.M., Andreini D., Pontone G., Alkadhi H., Hausleiter J., Zimmermann E. (2024). The Effectiveness of Coronary Computed Tomography Angiography and Functional Testing for the Diagnosis of Obstructive Coronary Artery Disease: Results from the Individual Patient Data Collaborative Meta-Analysis of Cardiac CT (COME-CCT). Insights Imaging.

[34] Shreya D., Zamora D.I., Patel G.S., Grossmann I., Rodriguez K., Soni M., Joshi P.K., Patel S.C., Sange I. (2021). Coronary Artery Calcium Score-A Reliable Indicator of Coronary Artery Disease?. Cureus.

[35] Reeh J., Therming C.B., Heitmann M., Højberg S., Sørum C., Bech J., Husum D., Dominguez H., Sehestedt T., Hermann T. (2019). Prediction of Obstructive Coronary Artery Disease and Prognosis in Patients with Suspected Stable Angina. Eur. Heart J..

[36] Hausleiter J. (2009). Estimated Radiation Dose Associated With Cardiac CT Angiography. JAMA.

[37] Halliburton S.S., Abbara S., Chen M.Y., Gentry R., Mahesh M., Raff G.L., Shaw L.J., Hausleiter J. (2011). SCCT Guidelines on Radiation Dose and Dose-Optimization Strategies in Cardiovascular CT. J. Cardiovasc. Comput. Tomogr..

[38] Li F., He Q., Xu L., Zhou Y., Sun Y., Wang Z., Xu Y., Yang Z., He Y. (2022). Diagnostic Accuracy of Subtraction Coronary CT Angiography in Severely Calcified Segments: Comparison Between Readers With Different Levels of Experience. Front. Cardiovasc. Med..

[39] McDermott M.C., Sartoretti T., Stammen L., Martens B., Jost G., Pietsch H., Gutjahr R., Schmidt B., Flohr T.G., Alkadhi H. (2024). Countering Calcium Blooming With Personalized Contrast Media Injection Protocols. Investig. Radiol..

[40] Langguth P., Wolf C., Sedaghat S., Huhndorf M., Frank J., Both M., Jansen O., Salehi Ravesh M., Lebenatus A. (2023). Clinical Value of Using Heart Rate Variability Biofeedback Before Elective CT Coronary Angiography to Reduce Heart Rate and the Need for Beta-Blockers. Appl. Psychophysiol. Biofeedback.

[41] Obed M., Gabriel M.M., Dumann E., Vollmer Barbosa C., Weißenborn K., Schmidt B.M.W. (2022). Risk of Acute Kidney Injury after Contrast-Enhanced Computerized Tomography: A Systematic Review and Meta-Analysis of 21 Propensity Score–Matched Cohort Studies. Eur. Radiol..

[42] Nicoli F., Mollace R., Allieri F., Frittella S., Collaku E., Lo Monaco M., Licastro M., Nudi A., Agati G., Brusamolino M. (2026). Stress Myocardial CT Perfusion Evaluation of Myocardial Blood Flow (MBF) Mean Value, and Correlation of MBF Defect and Main Risk Factors. Eur. Heart J. Cardiovasc. Imaging.

[43] Cacciatore S., Spadafora L., Bernardi M., Galli M., Betti M., Perone F., Nicolaio G., Marzetti E., Martone A.M., Landi F. (2023). Management of Coronary Artery Disease in Older Adults: Recent Advances and Gaps in Evidence. J. Clin. Med..

[44] Shaw L.J., Bugiardini R., Merz C.N.B. (2009). Women and Ischemic Heart Disease. J. Am. Coll. Cardiol..

[45] Kunadian V., Chieffo A., Camici P.G., Berry C., Escaned J., Maas A.H.E.M., Prescott E., Karam N., Appelman Y., Fraccaro C. (2021). An EAPCI Expert Consensus Document on Ischaemia with Non-Obstructive Coronary Arteries in Collaboration with European Society of Cardiology Working Group on Coronary Pathophysiology & Microcirculation Endorsed by Coronary Vasomotor Disorders International Study Group. EuroIntervention.

[46] Gulati M., Levy P.D., Mukherjee D., Amsterdam E., Bhatt D.L., Birtcher K.K., Blankstein R., Boyd J., Bullock-Palmer R.P., Conejo T. (2021). 2021 AHA/ACC/ASE/CHEST/SAEM/SCCT/SCMR Guideline for the Evaluation and Diagnosis of Chest Pain: A Report of the American College of Cardiology/American Heart Association Joint Committee on Clinical Practice Guidelines. Circulation.

[47] Swahn E., Sederholm Lawesson S., Alfredsson J., Fredrikson M., Angerås O., Duvernoy O., Engström G., Eriksson M.J., Fagman E., Johansson B. (2024). Sex Differences in Prevalence and Characteristics of Imaging-Detected Atherosclerosis: A Population-Based Study. Eur. Heart J. Cardiovasc. Imaging.

[48] Tzimas G., Gulsin G.S., Everett R.J., Akodad M., Meier D., Sewnarain K., Ally Z., Alnamasy R., Ng N., Mullen S. (2024). Age- and Sex-Specific Nomographic CT Quantitative Plaque Data From a Large International Cohort. JACC Cardiovasc. Imaging.

[49] Dimitriadis K., Iliakis P., Pyrpyris N., Tsioufis K. (2024). Unravelling Gender Differences in Coronary Artery Disease: Are We Equal?. Clin. Res. Cardiol..

